# Increased Amplitude of Low-Frequency Fluctuation in Right Angular Gyrus and Left Superior Occipital Gyrus Negatively Correlated With Heroin Use

**DOI:** 10.3389/fpsyt.2020.00492

**Published:** 2020-07-03

**Authors:** Jing Luo, Ru Yang, Wenhan Yang, Chunmei Duan, Yuan Deng, Jun Zhang, Jiyuan Chen, Jun Liu

**Affiliations:** ^1^ Department of Radiology, The Second Xiangya Hospital, Central South University, Changsha, China; ^2^ Yunnan Institute for Drug Abuse, Kunming, China; ^3^ Hunan Judicial Police Academy, Changsha, China; ^4^ Hunan Drug Rehabilitation Administration, Changsha, China

**Keywords:** resting state fMRI, amplitude of low-frequency fluctuation (ALFF), heroin addicts, addiction, heroin use

## Abstract

Abnormal amplitude of low-frequency fluctuation has been implicated in heroin addiction. However, previous studies lacked consistency and didn’t consider the impact of confounding factors such as methadone and alcohol. Fifty-one heroin-dependent (HD) individuals and 40 healthy controls underwent resting-state functional magnetic resonance imaging. The ‘amplitude of low-frequency fluctuation’ (ALFF) value was calculated and support vector machine (SVM) classification analysis was applied to analyze the data. Compared with healthy controls, heroin addicts exhibited increased ALFF in the right angular gyrus (AG) and left superior occipital gyrus (SOG). A negative correlation was observed between increased ALFF in the right angular gyrus and left superior occipital gyrus and the duration of heroin use (*p*_1_=0.004, *r*_1_=-0.426; *p*_2_=0.009, *r*_2_=-0.361). Moreover, the ALFF in the right AG and left SOG could discriminate the HD subjects from the controls with acceptable accuracy (Acc_1_=64.85%, *p*_1_=0.004; Acc_2_=63.80%, *p*_2_=0.005). HD patients showed abnormal ALFF in the brain areas involved in semantic memory and visual networks. The longer HD individuals abused heroin, the less the ALFF of associated brain regions increased. These observed patterns suggested that the accumulative effect of heroin’s neurotoxicity overpowered self-recovery of the brain and may be applied as a potential biomarker to identify HD individuals from the controls.

## Introduction

Heroin addiction is a chronic, relapsing brain disease characterized by addicts’ compulsive heroin seeking and consuming in spite of serious negative consequences (1). Over the past decades, various neuroimaging studies have revealed extensive structural and functional disruption in heroin-dependent (HD) individuals. Structural MRIs have revealed the impaired white matter integrity within the right frontal sub-gyral, corpus callosum, thalamic radiation, and inferior longitudinal fasciculus (2, 3), diminished regional homogeneity within the bilateral medial orbitofrontal cortex (OFC) and bilateral cuneus (4), and the reduced gray matter volume within the precuneus, cuneus, and right dorsolateral prefrontal cortex (DLPFC) (5, 6). Functional MRIs (fMRI), a classic method for assessing hemodynamic changes after increased neural activity, have been widely applied in neuroimaging with its merits of ever-increasing availability, its noninvasive nature, and its relatively high spatiotemporal resolution (7). Resting-state fMRI could explore the correlation of spontaneous neural excitation activity between different brain regions under rest (subjects didn’t undertake any language, cognitive, or motor tasks) (8). Resting-state fMRI disclosed increased functional connectivity (FC) between the anterior cingulate cortex (ACC) and nucleus accumbens and between the OFC and amygdala, and reduced FC between the ACC and prefrontal cortex (PFC) and between the OFC and PFC (9).

In 1995, Biswal and his co-workers first found that the spontaneous Low-Frequency (0.01–0.08 Hz) Fluctuations (LFFs) in resting-state fMRIs were most frequently found between the right and left primary motor cortex (10). After that, amplitude of low frequency fluctuation (ALFF) was further improved by Zang et al. (11). It was represented as the square root of the power spectrum in a low frequency range (0.01–0.08 Hz), which could evaluate the brain’s physiopathological state by computing the regional intensity of spontaneous fluctuation in Blood Oxygenation Level Dependent (BOLD) signal at rest. Studies applied to various diseases such as attention deficit hyperactivity disorder (11), epilepsy (12), Parkinson’s (12, 13), Alzheimer’s disease with depression (D-AD) (14), schizophrenia (15), and somatic depression (16) suggested that ALFF was a reliable approach to explore regional spontaneous neural activity (SNA) in resting-state.

ALFF measurement was firstly applied on HD subjects by Jiang et al. to study heroin-use-related SNA alteration (17). They studied 24 HD subjects (all subjects were treated with methadone during abstinence) and 24 control nondrug-using (CN) subjects and found that, compared with controls, the HD group had abnormal ALFF in multiple regions. Moreover, increased ALFF in the bilateral parietal lobe had a significantly positive correlation with the methadone dose, which suggested reduced ALFF may arise from taking heroin and the increased ALFF in the bilateral parietal lobe from the methadone treatment. Subsequently, the relationship between disrupted local neural activity and its functional organization pattern in resting-state was studied by Wang et al. with 17 male HD individuals and 15 controls (18). Their studies demonstrated that the ALFF value of the right caudate was negatively correlated with heroin use and an abnormal lateral PFC-dorsal ACC connection in the HD group, which suggested an altered balance between local neuronal assemblies’ activity. However, these studies above had some flaws. 1) Participant sample sizes were too small. The sample size of participants were less than fifty (HD individuals <30). Chen et al. assessed test-retest reliability and replicability of resting-state fMRI and found repeatability of studies with small sample sizes (<80) was low (19). 2) Participant samples were heterogenous. Alcohol abuse of participants wasn’t matched. A previous study demonstrated the degree to which alcohol use affected the reduction of thalamic grey matter volume in opioid-dependent subjects (20). In Jiang et al’s study, the use of methadone complicated the interpretation of their results. Methadone plays a vital role in the destruction of white matter integrity and resting state FC in heroin users under Methadone maintenance treatment (MMT) (21–24). In Wang et al’s study, all participants were male, which cannot comprehensively reflect ALFF differences in HD individuals. Therefore, these flaws above limited the reliability of these results.

To avoid the flaws from previous research, relatively larger (51 HD subjects and 40 CN subjects) and more homogeneous (unmedicated, cigarette, and alcohol abuse matched with professional questionnaire) samples were collected in the current study to investigate the ALFF differences in the whole brain between the HD and CN groups. In addition, to investigate potential biomarkers for heroin addiction, the support vector machine (SVM) classifier, which had already been successfully used in fMRI studies about heroin addiction (25, 26), was adopted on clusters that showed significant ALFF differences to discriminate HD subjects from healthy controls.

## Materials and Methods

### Subjects

We initially recruited 62 HD subjects (20-55 years old) and 44 age- and sex-matched healthy controls. Eleven HD subjects and four CN subjects were removed because of excessive head motion. Therefore, the present study included 51 HD subjects and 40 CN subjects. All HD subjects come from Pingtang Mandatory Detoxification in Changsha City, Hunan Province. All their diagnoses were confirmed with the fifth edition of Diagnostic and Statistical Manual on Mental Disorders (DSM-V) and after that had accepted a short-term (<6 months) (27) compulsory abstinence. During abstinence, the participants were treated without Methadone, only with education and physical exercise. Inclusion criteria included: ranging in age from 20 to 55 years old, receiving at least an elementary school education, being right-handed, and having no history of neurological and psychiatric disease other than drug addiction. Exclusion criteria for all subjects included: head trauma history, other substance use except nicotine and alcohol in the past 5 years, and contraindications to MR scanning. During the MRI examination interval, Fagerstrom Test for Nicotine Dependence (FTND) and Alcohol Use Disorders Identification Test (AUDI) were obtained for all participants (28, 29). The history of heroin use, including the duration of heroin use, dosage of heroin use, and abstinence periods, were also recorded from HD individuals.

This study was approved by the Ethics Committee of the Second Xiangya Hospital, Central South University. Informed consent was also obtained from each subject.

### MR Imaging Acquisition

All MRI examination data were obtained on a 3T Siemens Skyra MRI scanner (Magnetom Skyra, Siemens, Germany) with a 32-channel head coil. During the scans, participants were instructed to remain still, keep their eyes closed, and not think of anything in particular. The scanning sessions included: (i) localization; (ii) T1-weighted three-dimensional magnetization-prepared rapid acquisition with gradient echo (3D MPRAGE) (176 sagittal slices, slice thickness=1 mm, gap=0 mm, field of view (FOV)=256 mm×256 mm, repetition time (TR)=1450 ms, echo time (TE)=2.03 ms, inversion time (TI)=900 ms, flip angle=30°, and voxel size=1×1×1mm^3^); and (iii) resting-state fMRI sessions (36 axial slices, thickness=4 mm, FOV=220 mm×220 mm, TR=2000 ms, TE=30 ms, flip angle=80°, and 225 volumes). Participants stayed supine with foam padding between their head and the coil to minimize head movements.

### Data Processing and ALFF Calculation

Image data preprocessing was performed with Statistical Parametric Mapping (SPM 8) (SPM8, http://www.fil.ion.ucl.ac.uk/spm) and GRETNA (30). We firstly discarded the initial 10 scan volumes to allow for steady-state magnetization and then corrected for slice timing and head motion. We excluded participants whose head motion exceeded 2 mm or rotation exceeded 2°. After the resting state image registered with the T1 structure image, all images were reoriented into the AC-PC axis and then spatially normalized to Montreal Neurological Institute (MNI) space with the diffeomorphic anatomical registration through the exponential lie algebra (DARTEL) registration method (31). All images were smoothed using a 6mm half-height full-width Gaussian kernel. After that, linear detrending was conducted and covariates including white matter, cerebrospinal fluid, and 24 head movement parameters were subsequently removed.

The ALFF analysis was carried out with the Data Processing Assistant for Resting-State fMRI (DPABI, 2.3, Advanced edition) (32). The filtered time series of each voxel was transformed into the frequency domain with a Fast Fourier Transform and the power spectrum was then obtained. By measuring the square root of the signal across 0.01-0.08 Hz for each voxel (11), we obtained ALFF values. To reduce the influence of individual variation in ALFF values, the ALFF of each voxel was further divided by the global mean of ALFF values for each participant within the default brain mask from the DPABI. This made a standardized whole-brain ALFF map.

### Statistical Analysis

We performed two-sample *t*-tests to assess the differences in age, years of education, cigarette smoking, and alcohol use, and a chi-square test to assess the difference in gender between the HD and CN group with SPSS (version 22.0). The significance level was set at *p<*0.05. Two-sample *t*-tests were performed to compare ALFF data between the HD and CN group with DPABI to find significantly different brain regions. The significance level was set at *p<*0.001, cluster size*>*119 voxels (Gaussian random field corrected (GRF)).

### Correlation Analyzes

To identify the relationship between ALFF and history of heroin use, the average ALFF values of all abnormal clusters detected by group comparisons were extracted separately in every HD subject, and then partial correlation was applied to reveal the relationships between these ALFF values and the duration of heroin use, dosage of heroin use, and abstinence periods. Covariables included age, gender, years of education, smoking, and drinking. Significance levels were set at *p<*0.05 (two-tailed).

### Support Vector Machine (SVM) Analysis

To test whether the ALFF values of significant clusters could highlight potential biomarkers to identify HD individuals from the controls, SVM was conducted with matlab. A five-fold cross-validation method was used to conduct the SVM. The Classification Learner Tool svmtrain and svmclassify function in MATLAB was performed to classify ALFF values of the nine sub-ROIs using 10-fold cross validation. (https://stackoverflow.com/questions/13804833/using-matlab-svmtrain) The SVM kernel used was linear. This framework applies the whole data set split into 10 folds/subsets randomly; each subset consisted of 1/10 HD subjects and 1/10 healthy controls. One subset was used as test dataset, and the remaining nine subsets were used as a training dataset. The training dataset was used to train a classifier, which was then used to predict the test dataset. The training set and the test set are independent for each cross-validation. Accuracy, sensitivity, and specificity were computed to quantify the cross-validated prediction performance of these classifiers. After that, a permutation test with 1000 permutations was applied to test the significance of the classification results.

## Results

### Demographics and Clinical Characteristics of the Participants

Fifty-one HD subjects and 40 CN subjects were included in this study. We performed two-sample *t*-tests and chi-square tests to assess the demographics and clinical characteristics differences between the HD group and CN group. The significance level was set at *p<*0.05. There is no significant difference between the HD group and CN group in age (mean±SD) (42.90 ± 6.49 for HD group; 41.85 ± 8.13 for CN group; *t*=0.686, *p*=0.494), gender (36 male and 15 female for HD group; 28 male and 12 female for CN group; *χ^2^*=0.007, *p*=0.951), the years of education (9.33 ± 2.20 for HD group; 9.98 ± 1.84 for CN group; *t*=1.477, *p*=0.143), Fagerstrom Test for Nicotine Dependence (6.10 ± 2.21 for HD group; 5.53 ± 2.63 for CN group; *t*=1.128, *p*=0.262), or Alcohol Use Disorders Identification Test (1.50 ± 3.57 for HD group; 3.25 ± 4.39 for CN group; *t*=1.128, *p*=0.157) as shown in **Table 1**.

**Table 1:** Demographic information and characterization.

	HD(n=51)		CN(n=40)		*p*
Age (years)	42.90±6.49		41.85±8.13	*t*=0.686	0.494^a^
Gender (male/female)	36/15		28/12	*χ^2^*=0.007	0.951^b^
Education (years)	9.33±2.20		9.98±1.84	*t*=-1.477	0.143^a^
FTND	6.10±2.21		5.53±2.63	*t*=1.128	0.262^a^
AUDI	1.50±3.57		3.25±4.39	*t*=-1.573	0.120^a^
Duration of heroin use (years)	15.65±7.92		N/A	N/A	N/A
Dosage of heroin use (g/day)	1.25±3.85		N/A	N/A	N/A
Abstinence periods (days)	26.73±28.56		N/A	N/A	N/A

^a^Two-sample *t*-test; *b* Chi-square test. Significance level was set at *p*<0.05. There are no statistically significant differences between the HD and CN group based on demographic information and characterization; HD, heroin-dependent; CN, control nondrug-using; FTND, Fagerstrom Test for Nicotine Dependence; AUDI, Alcohol Use Disorders Identtification Test; N/A, not applicable.

**Figure 1:**
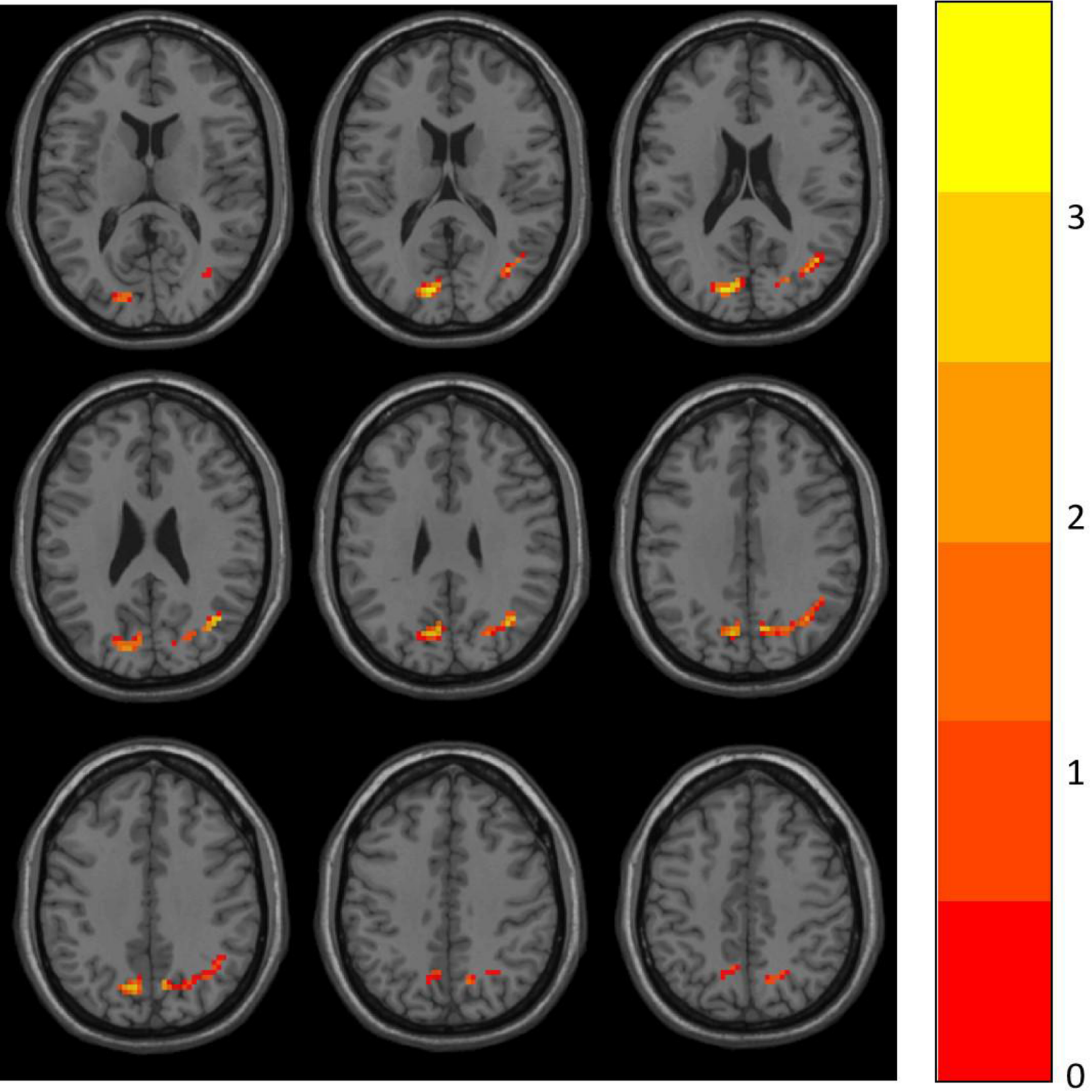
ALFF differences between the HD and CN group. The differences map at the given threshold (*p*<0.001, cluster size>119 voxels, Gaussian random field corrected) was shown. Blue indicates HD individuals had decreased ALFF compared with controls and the red indicates the opposite.

Two-sample *t*-tests were performed to compare ALFF data between the HD and CN group with DPABI. Group differences are shown in **Table 2** and **Figure 1**. In comparison with the CN group, the significant ALFF increases in the HD group were in the right angular gyrus (AG) and left superior occipital gyrus (SOG). No significant decrease cluster was found.

**Table 2:** Regions with increased ALFF in HD group compared with CN group.

Brain region (AAL)	Peak *t*-value	Cluster Size (voxels)	Peak MNI Coordinates		
			X	Y	Z
Occipital_Sup_L	3.7223	156	-12	-81	21
Angular_R	3.6889	146	39	-63	27

Statistical threshold was p<0.05 corrected for multiple comparisons by GRF. A combination threshold of voxels’ p<0.005 and cluster size>119 voxels was considered significant; Coordinates are located in MNI space; L, left hemisphere; R, right hemisphere; AAL, Anatomic-Automatic-Labeling template.

### Correlation Analyzes

**Figure 2:**
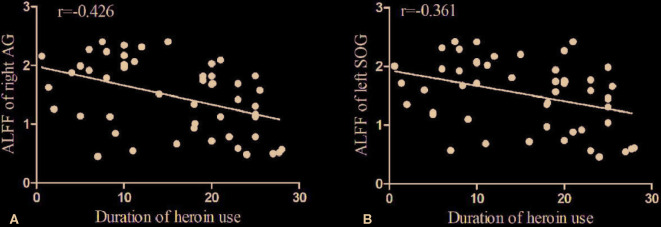
Bivariate scatter plots. A shows the negative correlation between the duration of the heroin use and the ALFF of the right AG (*p*=0.004, *r*=-0.426), and B the negative correlation between the duration of heroin use and the ALFF of the left SOG (*p*=0.009, *r*=-0.361).

Partial correlation was performed to assess relationships between above-significant differences ALFF values and the duration of heroin use, dosage of heroin use, and abstinence periods in HD individuals. Covariates included age, gender, years of education, smoking, and drinking. The length of duration of heroin use in HD subjects is significantly negatively correlated with ALFF in the right AG as shown in **Figure 2A** (*p*=0.004, *r*=-0.426). The length of duration of heroin use in HD subjects is also significantly negatively correlated with ALFF in the left SOG as shown in **Figure 2B** (*p*=0.009, *r*=-0.361).

### SVM Results

The SVM results were conducted with the five-fold cross-validation. The average accuracy for ALFF values in right AG and left SOG were 64.85% and 63.80%. The average sensitivity were 60.94% and 67.21%. The average specificity were 70.82% and 58.30%. The SVM classifier was then validated with a permutation test. After running1000 times with a 95% confidence interval, the average accuracy of the right AG and left SOG were 0.2747–0.6043 (*p* =0.004) and 0.2067 - 0.6044 (*p* =0.005).

## Discussion

In the present study, we compared ALFF between the abstinent HD group and the CN group and revealed that the HD group showed significantly increased ALFF in the right AG and left SOG. In addition, in the HD group, the length of duration of heroin use was significantly negatively correlated with the ALFF values of right AG and left SOG. Meanwhile, increased ALFF in the right AG and left SOG allowed for correct identification of the HD individuals from the controls with relatively high sensitivity, specificity, and accuracy.

The AG plays an important role in semantic memory (33, 34), which is involved in a series of tasks from single word recognition (35) to sentence-level comprehension (36). Semantic memory refers to a major division of long-term memory that includes knowledge of facts, events, ideas, and concepts. Little is known about the effects of chronic and excessive heroin consumption on the organization or extent of the pre-existing semantic networks. Previous studies used semantic memory association tasks to assess the implicit cognition of substance abusers (37). Implicit means that it cannot be recognized by introspection. It is a trace of people’s past experiences. Implicit cognition regulates people’s emotions, cognition, and reactions to likes and dislikes in society. Positive comments on drugs increased the likelihood of drug abuse, while negative evaluations increased the likelihood of avoiding drugs. The researchers believed that the current memory activation mode often dominated human behavior, and this activation mode was implicit. In other words, this activation mode was at a relatively spontaneous level. The experience of taking drugs many times will make it easier for people to establish and consolidate related memory connections. The next time they encountered drug-related cues, the idea of the drug-using experience in the addict’s brain will be automatically activated, which then triggers a memory-based concept-linked reaction. Therefore, researchers can use the semantic memory association task to assess the strength of drug-related memory connections between addicts. Previous research found that the results of the semantic memory association task can effectively predict alcohol and cannabis abuse in high-risk adolescents (38). A study found that, compared to the control group, cannabis and other drug abusers were more likely to have positive associations with clue topics, but non-abusers’ associations with drugs were usually harmful or negative. After receiving drug treatment, drug abusers will have more negative associations with the consequences of drug abuse (39). The above research showed that the research method of semantic memory association tasks involved in spontaneous and drug-related cognition provided individual differences’ indicators of associative memory. These indicators could help predict corresponding behaviors, including relapse. An early observational study (in the form of self-rating and observer-rating questionnaires) demonstrated that opiate addicts showed an increase in semantic affective memory (40). Semantic memory and novel semantic encoding impairment could result from alcohol dependence, which implied that specific impairment in category and feature learning may reflect a genuine deficit of new learning in the semantic memory (41, 42). In addition, angular gyrus is part of the default mode network, which is impaired in heroin addicts (43, 44). The increased ALFF in this region observed in our study was consistent with the previous report (17). In the present study, abnormal ALFF observed in the right angular gyrus may reveal pathological activation of semantic memory. After short-term withdrawal, heroin addicts will have more associations with drug-related cues, but whether this association was positive or negative remained unclear because of a lack of semantic memory associative tasks in our current study. To the best of our knowledge, there is no more research about the semantic memory of HD individuals in the literature. Therefore, further neuroimaging research is needed to clarify the relationship between heroin addiction and semantic memory.

The ALFF was also increased in the left SOG in our study, which is consistent with previous studies (18). The left SOG was an important part of the dorsal extrastriate cortex involved in higher level visual association processes (45). Many neuroimaging studies reported that the activity in visual brain regions was significantly linked to heroin-related cues exposure, a therapeutic effect, and prediction of relapse (46–49). Our result that the ALFF of the visual related brain cortex of the HD group increased implied that drug cue-induced craving, which is one of the most stable elements that leads to continued consumption and relapse across substances (50), is vital among HD individuals after short-term abstinence.

The SVM results indicated that increased ALFF in the right AG and left SOG can be used for differentiating the HD individuals from the controls with a relatively high sensitivity, specificity, and accuracy. Although previous studies suggested that a sensitivity of less than 70% is not so excellent (51), significance of the classification outcomes suggested that the result is acceptable in the present study. Hence, we inferred that increased ALFF in the right AG and left SOG can be used as a potential biomarker to identify the HD individuals from the controls.

In the current study, we first found that the length of duration of heroin use is significantly negatively correlated with the increased ALFF values of the right AG and left SOG. Previous studies only demonstrated that decreased ALFF in the caudate was in negative correlation to both the duration of the heroin use and the heroin daily dosage, and average ALFF in the elevated regions may respond to the effectiveness of methadone **(** 18). In our study, the increase in ALFF value could rule out the effect of treatment because the HD subjects didn’t receive methadone maintenance treatment. Interestingly, the longer HD individuals abuse heroin, the less the ALFF in the right AG and left SOG increase. Since increased ALFF may be due to the compensatory response and part recovery of associated brain function after withdrawal of heroin (17, 18), we speculated that the effect of heroin’s neurotoxicity overpowered self-recovery of the brain after short-term abstinence. Further study could explore ALFF changes of HD individuals after long-term abstinence.

### Advantages and Limitations

The current study explored HD subjects’ brain ALFF changes after short-term abstinence. To the best of our knowledge, this is the first study to demonstrate that the length of duration of heroin use is negatively correlated with the ALFF values of specific brain regions which show increased ALFF with respect to control. ALFF changes on semantic memory and visual network regions reveal that these areas play vital roles during heroin addiction and abstinence. This study provided a new perspective for understanding the pathological changes of ALFF in short-term abstinence HD individuals. Finally, ALFF values in the right AG and left SOG may be used as a potential biomarker to identify HD individuals from the controls.

There are some limitations in the current study. 1) This is a cross-sectional study, in which the neuroimaging data of HD subjects before abstinence was not obtained. Therefore, the causal relationship of abnormal ALFF values and the abstinence status could not be determined. 2) Some of the subjects have experienced no less than one period of abstinence. Repeated abuse and abstinence may affect the stability of ALFF in different brain regions. Further study with a first abstinence subgroup would help to solve this problem. 3) There are more men in compulsory detoxification, so the proportion of gender in the present study is not very even. Further study could recruit more women from HD individuals.

## Data Availability Statement

The datasets analyzed in this article are not publicly available. Requests to access the datasets should be directed to junliu123@csu.edu.cn.

## Ethics Statement

The studies involving human participants were reviewed and approved by Ethics Committee of the Second Xiangya Hospital, Central South University. The patients/participants provided their written informed consent to participate in this study.

## Author Contributions

JL, WY, JZ, and JL conceptualized and designed the research. JL, WY, JZ, and JC performed the experiments. JL and RY undertook the statistical analysis. JL and RY wrote the first draft of the manuscript. JL, RY, and JL contributed to the final manuscript. CD and YD provided funding support. All authors contributed to the article and approved the submitted version.

## Funding

Funding information: National Natural Science Foundation of China, Grant Number: 61971451. Natural Science Foundation of Hunan Province, China, Grant Number: 2015JJ4081. National Key Research and Development Program of China, Grant number: 2016YFC0800908. National Natural Science Foundation of China, Grant number: U1502225.

## Conflict of Interest

The authors declare that the research was conducted in the absence of any commercial or financial relationships that could be construed as a potential conflict of interest.
